# Sarcopenia and its associated factors among hip fracture patients admitted to a North African (Egyptian) Level one trauma center, a cross-sectional study

**DOI:** 10.1186/s13018-025-05841-w

**Published:** 2025-05-13

**Authors:** Dalia G Mahran, Ahmed A. Khalifa, Abdelhafeez Hamdi Abdelhafeez, Osama Farouk

**Affiliations:** 1 Department of Public Health and Community Medicine, Faculty of Medicine, Assiut University, Assiut, Egypt; 2 Department of Family Medicine, Faculty of Medicine, Assiut University, Assiut, Egypt; 3 Orthopaedic Department, Qena Faculty of Medicine and University Hospital at South Valley University, Qena, Egypt; 4 Orthopaedic Department, Assiut University Trauma Hospital, Assiut, Egypt

**Keywords:** Sarcopenia, Hip fracture, Osteoporosis, Skeletal muscle mass

## Abstract

**Objectives:**

The study's objectives were to assess the sarcopenia prevalence in hip fracture patients admitted to a North African (Egyptian) level one specialized trauma unit and to evaluate factors associated with sarcopenia.

**Methods:**

This was an analytic, cross-sectional study where patients who were admitted with low-energy hip fractures and managed surgically were included. Assessment was performed using the SARC-F questionnaire, InBody device assessments (skeletal muscle mass (SMM), Fat mass, nutritional status (total water, protein, and minerals)), handgrip strength, and body mass index (BMI). Sarcopenia was diagnosed based on the revised European Working Group on Sarcopenia in Older People criteria (EWGSOP2).

**Results:**

The patients' mean age was 68 ± 8.3 years; 51.9% were females. The mean SMM was 24 ± 4.5 kg, while the mean handgrip strength was 20.55 ± 7.66 kg, sum SARC-F score was normal in 115 (85.2%) patients and abnormal in 20 (14.8%). Based on the EWGSOP2 criteria, 23 (17%) patients had sarcopenia, and 112 (83%) did not. The two groups were comparable regarding age and sex (*p* = 0.907 and 0.623, respectively). Sarcopenic patients had significantly lower values in BMI (21.9 vs. 25.9 kg/m^2^, *p* < 0.001), SMM (14.8 vs. 23, *p* < 0.001), BMR (*p* < 0.001), Fat mass (18.8 vs. 24.3, *p* = 0.003), and handgrip strength (16 vs. 20 kg, *p* = 0.034), however the sum SARC-F score ≥ 4 points, was higher in sarcopenic group (30.4% vs. 11.6%, *p* = 0.046). SMM, BMR, and fat mass showed large effect sizes (≥ 5), while the handgrip strength showed a medium effect size (0.3). There was a significant negative correlation between patients' age and handgrip strength (*r* = -0.394, *p* < 0.001), and a significant positive correlation between BMI and the SMM (*r* = 0.210, *p* = 0.014). Univariate logistic regression analysis revealed that the patient’s BMI, fat mass, total water, protein, minerals, and the sum of SARC-F were significantly associated with sarcopenia development. However, on multivariate logistic regression analysis, two factors kept a significant association: the protein levels as a marker of nutritional reserve (OR = 0.044, 95%CI = 0.008 to 0.235, *P* < 0.001) and the sum SARC-F ≥ 4 points as a proxy for functional decline (OR = 6.365, 95%CI = 1.272 to 31.854, *P* = 0.024).

**Conclusion:**

The sarcopenia prevalence in our hip fracture patients was 17%, where BMI, fat mass, and nutritional status had a significant negative association; on the other hand, the sum of SARC-F (≥ 4 points) had a significant positive association. However, after multivariate analysis, only protein levels and the sum of SARC-F remained significantly associated with sarcopenia.

**Supplementary Information:**

The online version contains supplementary material available at 10.1186/s13018-025-05841-w.

## Background

Sarcopenia is a condition in which individuals develop progressive loss of muscle mass, function, strength, resulting in or associated with low physical performance [1–5]. It leads to an increased risk of falls and subsequent various types of injuries, including hip fractures [4, 6]. The literature reports its incidence varying from up to 29%, which might increase to 50% in individuals > 80 years old [7, 8].

On the other hand, hip fractures, including fragility fractures, which could be partially attributed to sarcopenia, are a rising worldwide healthcare concern [9–12]. In 2000, an estimated 1.6 million fractures occurred in individuals older than 50 [13], with an expected increase to 4.5 million by 2050 [14]; furthermore, the same increasing trend applies to the Egyptian population [15]. These fractures lower patients'life expectancy, increase mortality incidence, and have a socioeconomic impact on healthcare systems [16–19].

There is an alternating relationship between sarcopenia and hip fractures, where sarcopenia leading to increasing fall risk could lead to hip fractures. In contrast, patients treated for hip fractures (either surgically or non-surgically) are at an increased risk of developing sarcopenia attributed to long periods of recumbency, immobilization, and malnutrition [4, 20–22]. So, properly detecting and managing sarcopenia is paramount to decreasing the risk of further falls and fractures, ultimately improving hip fracture patients'quality of life [5, 23–25].

Studies evaluating the incidence and factors related to sarcopenia in hip fracture patients are rare in our area [26–28]; furthermore, to assess sarcopenia, most of these studies used the SARC-F score, which was considered by the European Working Group on Sarcopenia in Older People (EWGSOP2) as a screening tool [2].

The study's primary objective was to assess the prevalence of sarcopenia in hip fracture patients admitted to a North African (Egyptian) level one specialized trauma unit where the diagnosis was made based on the confirmatory criteria suggested by EWGSOP2 [2]. The study's secondary objective was to evaluate factors associated with sarcopenia in those patients.

## Methods

### Study design

This was an analytic, cross-sectional study carried out between November 2022 and September 2023 after obtaining approval from our institutional ethical committee and institutional review board (IRB approval no.:17101579); furthermore, we followed all ethical considerations according to Helsinki declarations. We followed STROBE guidelines for reporting the current study (Supplementary file 1) [29].

### Study setting

A North African (Egyptian) Level one trauma center (affiliated with a university teaching hospital).

The sample size for the current study was calculated using Epi info, version 7. Considering a 46% prevalence of sarcopenia in hip fracture patients as reported in the literature [30], a confidence level of 95% and power of 80%. Given that approximately 200 hip fracture patients (fulfilling the inclusion criteria) are admitted annually to our trauma hospital, the calculated sample size was 132. To guard against missing data, we increased the sample by 15% to a total of 150 hip fracture patients.

### Study participants

We included patients aged ≥ 50 years old who presented to our trauma unit with an isolated traumatic hip fracture (no other associated skeletal injuries) resulting from a relatively low-energy trauma (a fall from a standard height), where the decision was to treat the fracture surgically, and patients agreed to participate in the study. We excluded patients aged > 80 years old who sustained a pathological fracture, those known to have dementia or neurological conditions, those known to have muscle disease, uncooperative patients, and those having concomitant other injuries.

Before starting final patient inclusion and data collection, we carried out a pilot study on 15 participants (who were not included in the study) to fulfill the following purposes: 1- To test the feasibility and simplicity of the used questionnaires and to improve and clarify any difficulties or ambiguities. 2- Estimating the time needed to collect data. 3- Detect the difficulties that may arise and how to deal with them. 4- To ensure the familiarity of the rehabilitation department personnel with the assessment being performed (such as handgrip strength and InBody measurements).

**Fig. 1:**
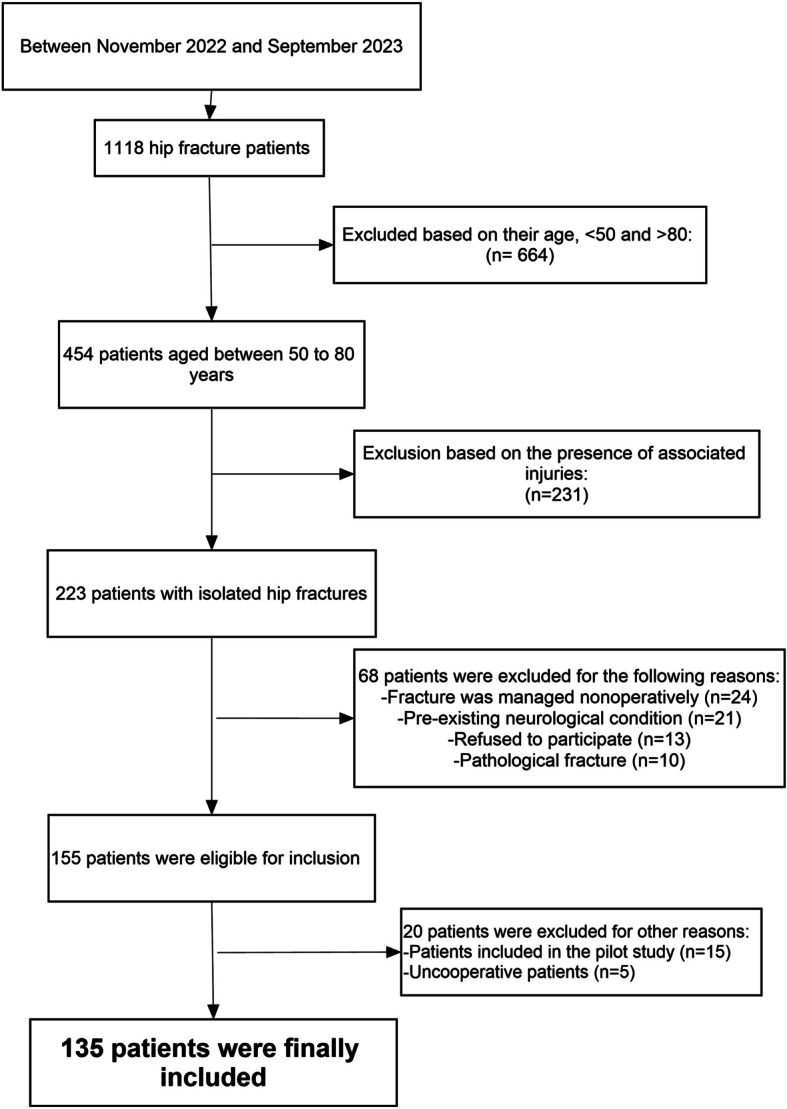
A flowchart showing the patient recruitment and inclusion process

After applying the aforementioned inclusion and exclusion criteria, 135 patients were eligible for final inclusion (details of the patient selection process are demonstrated in Fig. 1).

According to our institution protocol [31], the pathway of patients with hip fractures is formed of the following: 1- evaluation upon arrival, which usually includes history, physical examination, and radiological assessment. 2- admission and deciding on the best management option, whether operatively or non-operatively. Usually, operative intervention (open reduction and internal fixation for intertrochanteric fractures or hip replacement (hemi or total) for femoral neck fracture cases) was performed as early as possible if the patient's general condition allows. 3-Post-operative care and rehabilitation protocol are usually performed under a trained physiotherapist's supervision. Patients were mobilized from the first postoperative day, and the weight-bearing status was adjusted according to the injury type and its management. 4-Discharge and follow up protocol: Patients were discharged at least on the third postoperative day to their residence or a health facility if needed. Follow-up visits are scheduled for wound evaluation, radiographic assessment, patient function progression, and detection of any complications.

### Outcomes variables evaluation and Data collection

All the outcome parameters were collected during the hospital stay (by one of the authors (A.H.A.) who was not involved in the final data analysis) as follows:

### A-Patients self-administered questionnaire (supplementary file 2)

A pre-designed, semi-structured questionnaire was used in a previous study on patients from the same population [32]. The questionnaire was delivered in the patient's mother tongue language and was divided into four sections as follows:

*First,* the patient's demographic data were included, such as name (which was kept anonymous during data analysis), age, sex, residence, ID number, and contact information. *Second,* include questions about the history of chronic diseases, previous surgeries, previous fractures, previous medication, or previous hospitalization. *Third,* questions about muscle health, such as muscle strength, were included by asking the patient about his/her preinjury status and ability to walk independently, use walking aids, stand up from a chair, and climb stairs. *Fourth,* it included questions about dietary and special habits such as smoking, coffee, soda, or tea drinking or milk, cheese, or yogurt eating.

The questionnaire was delivered to the patient in person in Arabic (the native language for all patients) by one of the authors (who was not involved in data analysis) on the first day after admission and before surgical intervention.

*B-SARC-F score:* The SARC-F questionnaire comprises five Sects. (0 to 2 points for each section): strength, walking assistance, rising from a chair, stairs climbing, and falls. The final score ranges from 0 to 10, where a score of ≥ 4 predicts sarcopenia and poor outcomes [33–35].

*C-Investigations:* These were carried out postoperatively during the hospital stay (with the assistance of rehabilitation department personnel).

1-InBody device test: this was carried out in the rehabilitation department before patient discharge (usually on the second or third postoperative days, based on the fact that the patient's muscle mass remains stable up to ten days postoperatively [36]). InBody devices utilize direct segmental measurement bioelectrical impedance analysis (DSM-BIA) to accurately estimate body composition. This process is accomplished by sending multiple electrical currents through the body, resulting in up to six different impedance readings for the trunk and each of the four limbs. The InBody test results in accurate evaluation outputs of the body composition, including body fat mass, skeletal muscle mass (SMM), lean body mass, and percent body fat [37, 38].

2-Anthropometric measurement: Weight, height, and body mass index (BMI) were estimated using Peterson et al.'s methods [39]. Weight: measured while the participant is standing on paired legs and wearing light clothes, using the InBody device. Height: it was measured while the participants were standing on paired legs, using a stadiometer while the patients were upright. Assistant nurses in the rehabilitation room took measurements of every patient's weight and height. The BMI was determined based on weight and height (Kg/m^2^) and was interpreted as underweight (< 18.5), normal healthy weight (18.5—24.9), overweight (25.0—29.9), and obese (≥ 30.0).

3- Handgrip strength: A digital hand dynamometer was used to evaluate isometric grip force [40, 41]. While the patient was seated, the limb to be evaluated (dominant side) was positioned as follows: shoulder abduction, elbow flexed to 90 degrees, and the forearm and wrist were kept neutral. The patient was asked to hold the dynamometer and smoothly apply maximum grip force, and this step was repeated three times; the average of the three readings was considered the final handgrip strength and presented in kilograms.

Sarcopenia was diagnosed according to the definition set by the EWGSOP2 [2], where the probability of sarcopenia is identified by low muscle strength diagnosed using handgrip strength assessment (the cutoff value was set at < 16 kg for females and < 27 kg for males), and the diagnosis was confirmed by using an additional criterion, which is the low muscle quantity or quality, where the cutoff values for SMM was set at < 15 kg for females and < 20 kg for males. Based on the abovementioned criteria, the included patients were divided into two groups: those who confirmed having sarcopenia and those who did not.

### Statistical analysis

All statistical calculations were performed using SPSS (statistical package for the social science; SPSS Inc., Chicago, IL, USA) version 22. Data were described as mean ± standard deviation (± SD), or median and range (according to the status of data normal distribution), frequencies (number of cases), and relative frequencies (percentages) when appropriate. Quantitative variables were compared using the Mann–Whitney U test, as the data were not normally distributed. For comparing categorical data, the Chi-square (χ2) test was performed, or the Fisher Exact test was used instead of Chi-square (χ2) when the expected frequency is less than 5. To estimate the differences magnitude between both groups, effect sizes were calculated for significant comparisons, where Cliff's Delta was used (as a non-parametric effect size measure), and the presented values are interpreted as follows: if < 0.1: Negligible effect (The difference is very small and not meaningful). 0.1 to < 0.3: Small effect (The difference is small but maybe meaningful). 0.3 to < 0.5: Medium effect (the difference is moderate and likely meaningful). ≥ 0.5: Large effect (the difference is significant and meaningful). For categorical data, the risk difference (RD) and odds ratio (OR) were calculated as effect size estimates. Post-hoc power calculations were performed using G Power software (version 3.1) based on the observed effect and sample sizes, with a significance level (alpha) of = 0.05. Correlation between various variables was done using the Pearson correlation test. Odds ratio (OR) with a 95% Confidence Interval (CI) and Logistic Regression were calculated to measure the different risk factors for sarcopenia development. A P-value is always two-tailed and set significantly at 0.05 level.

## Results

The mean age of the included patients was 68 ± 8.3 years; 51.9% were females, 59.3% presented with intertrochanteric fractures, and 40.7% presented with neck of femur fractures (all patients were treated surgically). Further details regarding medical comorbidities and nutritional habits are reported in Table 1. For the whole patients, the mean SMM was 24 ± 4.5 kg, having a median of 23.5 (ranging from 15.3 to 48.1), while the mean handgrip strength was 20.6 ± 7.7 kg, having a median of 20 (ranging from 10 to 50). Based on the EWGSOP2 criteria, 23 (17%) patients were confirmed to have sarcopenia, while 112 (83%) did not. However, according to the sum SARC-F score, 115 (85.2%) patients were normal, and 20 (14.8%) were abnormal (probable sarcopenia) (Table 1).

**Table 1:** Socio-demographic data of the studied participants

Variables			Total (*n* = 135)
*Baseline data*			
Age (years)			68 ± 8.3 (50–80)*, 69 (63–75)^†^
Sex^‡^	Male		65 (48.1%)
	Female		70 (51.9%), [Premenopausal: 3 (4.3%), Postmenopausal: 67 (95.7%)]
BMI (kg/m^2^)			25.8 ± 5.3*, 25.4 (13.6–37.6)^†^
BMI categories^‡^	Underweight (< 18.5)		9 (6.7%)
	Normal weight (18.5–24.9)		53 (39.3%)
	Overweight (25–29.9)		40 (29.6%)
	Obese (≥ 30)		33 (24.4%)
Smoking^‡^	Yes		28 (20.7%)
	No		107 (79.3%)
Medical comorbidities^‡^	DM		36 (26.7%)
	HTN		45 (33.3%)
Steroid intake^‡^	Yes		5 (3.7%)
	No		130 (96.3%)
Chronic diseases^‡^	Yes		27 (20%)
	No		108 (80%)
nutritional habits^‡^(daily consumption)	Coffee consumption		10 (7.4%)
	Tea consumption		111 (82.2%)
	Soda consumption		25 (18.5%)
	Milk		38 (28.1%)
	Cheese		38 (28.1%)
	Yogurt		23 (17%)
Fracture type^‡^	Intertrochanteric fracture		80 (59.3%)
	Neck of femur		55 (40.7%)
*InBody, DEXA, and SARC-F score measurement of the studied group*			
SMM (kg)			24 ± 4.5*, 23.5 (15.3–48.1)^†^
Fat mass (kg)			23.1 ± 8.7*, 23.2 (1.1–48)^†^
BMR			1275.8 ± 156.1*, 1257 (1025–2039)^†^
Nutritional status	Total water (L)		30.6 ± 5.2*, 28.9 (22.1–56)^†^
	Protein (Kg)		8.4 ± 1.5*, 8.2 (5.7–16.6)^†^
	Minerals (Kg)		2.9 ± 0.4*, 2.9 (1.9–4.5)^†^
Hand grip strength (kg)			20.6 ± 7.7*, 20 (10–50)^†^
SARC-F score ^‡^	Strength	Not at all	108 (80)
		Some difficulty	20 (14.8)
		Very difficult	7 (5.2)
	Assistance walking	Not at all	115 (85.2)
		Some difficulty	13 (9.6)
		Very difficult	7 (5.2)
	Rising from a chair	Not at all	115 (85.2)
		Some difficulty	16 (11.9)
		Very difficult	4 (3)
	Climbing stairs	Not at all	105 (77.8)
		Some difficulty	14 (10.4)
		Very difficult	16 (11.9)
	Falls	None	78 (57.8)
		1–3 times	29 (21.5)
		> 3 times	28 (20.7)
	Sum SARC-F	Normal (0—3 points)	115 (85.2%)
		Abnormal (≥ 4 points)	20 (14.8%)

^*^mean ± SD (range). †Median (IQR range). ‡ Number (percentage)

*SMM* skeletal muscle mass, *BMR* basal metabolic rate, *L* Liter, *Kg* kilogram

### Comparison Between Sarcopenic and Non-Sarcopenic Patients

The two groups (Sarcopenia vs. not) were comparable regarding age and sex (*p* = 0.907 and 0.623, respectively); furthermore, no differences were found between groups related to Smoking, Fracture type, Steroid intake, Chronic diseases, and nutritional habits (supplementary file 3). However, sarcopenic patients were significantly lower in BMI (kg/m^2^) (*p* < 0.001), nutritional status (*p* < 0.001), SMM (*p* < 0.001), Fat mass (*p* = 0.003), BMR (*p* < 0.001), and handgrip strength (*p* = 0.034), however, the sum SARC-F score ≥ 4 points, was significantly higher in the sarcopenic group (*p* = 0.046), except for falls components where p-value was 0.204. The effect size for the significant comparisons ranged from small to large, where SMM, BMR, and fat mass showed large effects, while the handgrip strength showed a medium effect. Post-hoc power calculations indicated that for most of the variables, the study was adequately powered (power ≥ 80%) to detect the significant differences between variables, except for the total water component of the nutritional status and the Sum SARC-F, which had a power of 65% (Table 2).

**Table 2:** Differences in Socio-demographic data, body composition, SARC-F score, and muscle performance between patients with sarcopenia and those without

Variable			No Sarcopenia (*n* = 112)	Sarcopenia (*n* = 23)	*P*-value	Effect size**	Post-Hoc Power analysis**
*Baseline data*							
Age (years)^†^			69 (50–80)	67 (57–80)	0.907^§^	NA	NA
Sex^‡^		Female	55 (49.1)	10 (43.5)	0.623^¶^	NA	NA
		Male	57 (50.9)	13 (56.5)			
BMI (kg/m^2^)^†^			25.9 (17.7—37.6) 95% CI: 23.18 to 28.62	21.9 (13.6—33.0) 95% CI: 16.04 to 27.76	** < 0.001**^§^	0.3*	85%
Nutritional status^†^	Total water		29.3 (25–56) 95% CI: 25.05 to 33.55	27.1 (22.1—38.2) 95% CI: 22.22 to 31.98	** < 0.001**^§^	0.2*	65%
	Protein		8.4 (6.5—16.6) 95% CI: 7.01 to 9.79	6.8 (5.7—8.9) 95% CI: 5.84 to 7.76	** < 0.001**^§^	0.4*	95%
	Minerals		2.9 (2.2—4.5) 95% CI: 2.59 to 3.21	2.5 (1.9—3.5) 95% CI: 2.01 to 2.99	** < 0.001**^§^	0.3*	85%
*body composition and muscle performance*							
SMM^†^			23 (14.2—46.1) 95% CI: 18.2 to 27.8	14.8 (13.3—19.8) 95% CI: 12.7 to 16.9	** < 0.001**^§^	0.6*	99%
Fat mass^†^			24.3 (2.4—48) 95% CI: 17.5 to 31.1	18.8 (1.1—28.3) 95% CI: 9.9 to 27.8	**0.003**^§^	0.5*	98%
BMR^†^			1276 (1056–2039) 95% CI: 1129.2 to 1422.8	1134 (1025–1397) 95% CI: 1011.6 to 1256.4	** < 0.001**^§^	0.6*	99%
Handgrip strength (kg)^†^			20 (10–50) 95% CI: 14.03 to 25.97	16 (10–30) 95% CI: 9.4 to 22.6	**0.034**^§^	0.3*	85%
*SARC-F score*^*‡*^							
Strength	Not at all		93 (83%)	15 (65.2%)	**0.019**^¶^	NR	NR
	Some difficulty		16 (14.3%)	4 (17.4%)			
	Very difficult		3 (2.7%)	4 (17.4%)			
Assistance walking	Not at all		99 (88.4%)	16 (69.6%)	**0.016**^¶^		
	Some difficulty		10 (8.9%)	3 (13%)			
	Very difficult		3 (2.7%)	4 (17.4%)			
Rising from a chair	Not at all		98 (87.5%)	17 (73.9%)	**0.020**^¶^		
	Some difficulty		13 (11.6%)	3 (13%)			
	Very difficult		1 (0.9%)	3 (13%)			
Climbing stairs	Not at all		90 (80.4%)	15 (65.2%)	**0.013**^¶^		
	Some difficulty		13 (11.6%)	1 (4.3%)			
	Very difficult		9 (8.0%)	7 (30.4%)			
Falls	None		67 (59.8%)	11 (47.8%)	0.204^¶^		
	1–3 times		25 (22.3%)	4 (17.4%)			
	> 3 times		20 (17.9%)	8 (34.8%)			
Sum SARC-F	Normal (0—3 points)		99 (88.4%)	16 (69.6%)	**0.046**^¶^	OR: 3.33^#^ RD: 0.188^#^	65%
	Abnormal (≥ 4 points)		13 (11.6%)	7 (30.4%)			

^†^Median (IQR range). ‡ Number (percentage)

^§^ Mann Whitney U test was used for comparison. Chi-square (χ2) test was used to compare categorical data. For effect size analysis: *Cliff's Delta (< 0.1: Negligible effect. 0.1 to < 0.3: 0.3 to < 0.5: Medium effect. ≥ 0.5: Large effect), # odds ratio (OR), and risk difference (RD). (** was carried out for variables with significant differences). The P-value is significant if ≤ 0.05 (indicated by bold numbers)

*SMM* skeletal muscle mass, *BMR* basal metabolic rate, *BMI* body mass index, *NA* not applicable, *NR* not required

### Correlation analyses

**Fig. 2:**
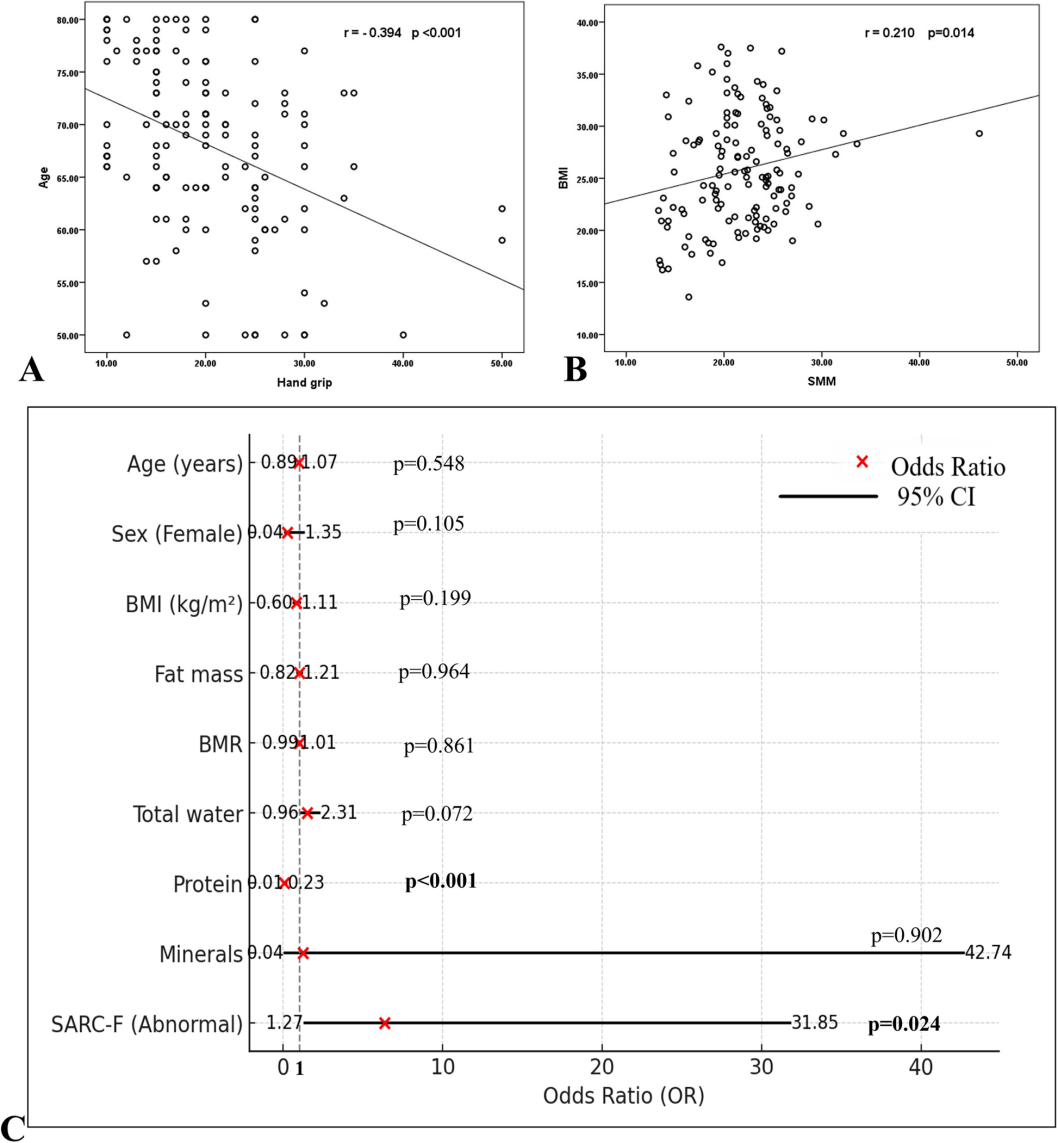
Scatter plot diagram showing the correlation between: **A** age and handgrip strength. **B** body mass index (BMI), and skeletal muscle mass (SMM). **C** Forest plot for the multivariate regression analysis results

Regarding correlation analysis, there was a significant negative correlation between patients'age and handgrip strength (*r* =—0.394, *p* < 0.001) and a significant positive correlation between BMI and the SMM (*r* = 0.210, *p* = 0.014) (Table 3, and Fig. 2 A & B).

**Table 3:** The correlation between muscle mass and hand grip and age, and BMI among the studied participants

		SMM	Handgrip strength
Age (*n* = 135)	r	−0.165 95% CI: −0.32 to 0.00	−0.394 95% CI:−0.53 to −0.24
	p	0.056	** < 0.001**
BMI (*n* = 135)	r	0.210 95% CI: 0.04 to 0.37	−0.165 95% CI: −0.32 to 0.00
	p	**0.014**	0.056

The p-value is significant if ≤ 0.05 (indicated by bold numbers), r = correlation coefficient

*BMI* body mass index, *SMM* skeletal muscle mass, *CI* confidence interval

### Regression Analyses for Sarcopenia-associated factors

Univariate logistic regression analysis to assess factors associated with sarcopenia among the studied patients revealed that the patient’s BMI, fat mass, BMR, total water, protein, minerals, and the sum SARC-F were significantly associated with the presence of sarcopenia. However, on multivariate logistic regression analysis, protein and the sum SARC-F remained significantly associated with sarcopenia, as we observed that for the protein levels, the odds ratio (OR) = 0.044, 95%CI = 0.008 to 0.235, *P* < 0.001; and patients with abnormal sum SARC-F (≥ 4 points) had an increased the risk of developing sarcopenia by about six times compared to patients with normal sum SARC-F (0–3 points) (OR = 6.365, 95%CI = 1.272 to 31.854, *P* = 0.024) (Table 4, Fig. 2C).

**Table 4:** Univariate and Multivariate logistic regression analysis for factors associated with developing sarcopenia among the studied participants

Variables		Univariate analysis			Multivariate analysis		
		OR	95% CI	*P*-value	OR	95% CI	*P*-value
Age (years)		1.008	0.95 to 1.07	0.785	0.972	0.89 to 1.07	0.548
Sex	Male	ref			ref		
	Female	1.254	0.51 to 3.1	0.623	0.238	0.04 to 1.35	0.105
BMI (kg/m^2^)		0.809	0.72 to 0.91	** < 0.001**	0.816	0.6 to 1.11	0.199
Fat mass		0.906	0.85 to 0.96	**0.002**	0.995	0.82 to 1.21	0.964
BMR		0.991	0.99 to 1	**0.001**	1.001	0.99 to 1.01	0.861
Total water		0.742	0.61 to 0.9	**0.003**	1.495	0.97 to 2.32	0.072
Protein		0.216	0.11 to 0.44	** < 0.001**	0.044	0.01 to 0.24	** < 0.001**
Minerals		0.031	0.01 to 0.19	** < 0.001**	1.248	0.04 to 42.74	0.902
Sum SARC-F	Normal (0—3 points)	ref			ref		
	Abnormal (≥ 4 points)	3.332	1.16 to 9.62	**0.026**	6.365	1.27 to 31.85	**0.024**

The p-value is significant if ≤ 0.05 (indicated by bold numbers)

*BMR* basal metabolic rate. *BMI* body mass index, *CI* Confidence interval; *OR* Odds ratio

## Discussion

The most important finding of the current study was that the prevalence of sarcopenia in Egyptian hip fracture patients was 17%, which did not differ between males and females. Sarcopenia was significantly associated with lower BMI, poorer nutritional status, reduced muscle mass and performance, and higher SARC-F scores (except for falls). Correlation analysis revealed a significant relationship between age, BMI, and muscle performance metrics. Logistic regression analysis identified low total protein and high SARC-F scores as significant associates with sarcopenia.

Outcomes, morbidity, and mortality after managing hip fractures, especially in the elderly, could be affected by various factors; sarcopenia has been proven to be a significant modifier leading to earlier mortality and poorer outcomes in those patients [42–45]. Based on these concerns, screening for sarcopenia in hip fracture patients became a concern for many authors from different countries [27, 30, 44, 46], aiming at offering better rehabilitation and nutritional programs for those proven to have sarcopenia with eventual improvement in the overall outcomes, quality of life, and mortality rates [47, 48].

We selected a lower cutoff age limit (50 years old) for some reasons: to capture as many patients with sarcopenia associated with isolated hip fracture as possible, where hip fracture risks increase after this particular age, especially in postmenopausal women (in the current study 95.7% of the females were postmenopausal) owing to the estrogen deficiency associated decline in bone mineral density [13, 14]. Furthermore, some previous studies used the same cutoff age value, allowing valid comparisons with their results [26, 27].

The EWGSOP2 criteria were selected in the current study for several factors, including but not limited to their comprehensive and widely accepted approach in diagnosing sarcopenia, where they provide a clear definition involving muscle mass and strength assessment with defined cut-off values for both males and females, leading to an accurate diagnosis [2]. Furthermore, these criteria were used in other studies on different populations, ensuring their reliability and suitability for comparing the obtained results with other studies [2, 44].

We evaluated the sarcopenia in patients during their hospital stay, which is considered a proper time to obtain reliable results. According to D'Adamo et al., there is no significant change in total body lean mass between three and 10 days after hip fracture operative management [36]. Evaluating the muscle mass shortly after surgery is beneficial, as some studies suggest that as time passes after surgery, there will be a greater decrease in the lower extremity and total body lean mass, where the loss was estimated to reach up to 9% and 5% from 10 days to 4 months post-injury [49–51].

Although dual-energy x-ray absorptiometry (DXA) scan evaluation was utilized in various studies [30, 46, 52], due to logistic and financial constraints, we could not get a DXA scan for our patients; instead, we relied on muscle mass and handgrip strength assessment for diagnosing sarcopenia [2]. McLester et al. reported the reliability of InBody testing as an alternative in the absence of DXA assessment [37]. Furthermore, Oyama et al. reported the comparability of assessing muscle mass using the InBody technique compared to CT-obtained measurements [53]. However, it is worth noting that the InBody assessment results might be affected by some factors in specific populations, such as edema, fluid imbalance, and severe obesity [54].

To add more clinical context to the significant comparisons in the current study, we evaluated the effect sizes, which ranged from medium to large (as shown in Table 2), showing that 80% of sarcopenic patients had lower SMM; furthermore, 75% and 80% of the no sarcopenia group had higher fat mass and BMR, respectively. However, handgrip strength showed the lowest effect size (0.3), indicating that only 65% of the no sarcopenia group had higher strength. This lower effect size could be attributed to the nature of handgrip strength evaluation; unlike other variables, handgrip strength relied partially on the patient contribution; furthermore, its assessment involved evaluating the quality and quantity of the muscle. A large effect size for the SMM confirms severe muscle depletion in sarcopenia patients, which empowers its diagnostic value; furthermore, it highlights further management options to prevent further muscle mass loss through proper nutrition and physical activity. The same applies to the BMR, where sarcopenic patients showed a metabolic slowdown, leading to increased frailty and deficient recovery; this should be managed by recommending protein supplementation to compensate for such an energy deficit. A 75% effect size associated with fat mass differences might indicate the body composition heterogeneity, where some sarcopenic patients might retain fat mass, which might be called “sarcopenic obesity”, where in such cases, the management protocol should be tailored per patient, ranging from weight management vs. nutritional support. Lastly, although hand grip strength is valuable for sarcopenia diagnosis, its lower effect size highlights the importance of considering its value in association with other variables.

Two studies evaluated the prevalence of sarcopenia in the Egyptian population, one by El-Miedany et al. [26], and the other by Sanad et al. [27]. In both studies, the authors used the SARC-F questionnaire for sarcopenia assessment, which is considered a screening test according to EWGSOP2. In contrast, besides using SARC-F in the current study, we relied on the confirmatory criteria for diagnosis (muscle mass and strength) per EWGSOP2, which suggests a more accurate sarcopenia prevalence estimation. The previous remark was noticed in the current study, where the sarcopenia prevalence was 14.8% according to SARC-F scores, which increased to 17% when applying the operational definition of sarcopenia according to EWGSOP2 based on muscle mass and power testing [2].

Furthermore, we reported lower sarcopenia prevalence (17%) compared to 59.5% and 29.3%, as reported by El-Miedany et al. [26], and Sanad et al. [27], respectively. Besides differences in the sarcopenia assessment tools, this discrepancy could be attributed to the fact that El-Miedany et al. [26], included 405 patients (121 males and 284 females) having a mean age of 70.1 ± 9.2 (the minimum age limit was 50 years), which was comparable to our included population; however, the authors did not exclusively include hip fracture patients; they also included patients attending the fracture clinic who presented with other fragility fractures (including spine and forearm fractures). On the other hand, the Sanad et al. study included 140 patients having a mean age of 68.3 ± 6.9, with the minimum age for inclusion being 60 years old [55], which is relatively higher than the age limit we set for the current study.

Sarcopenia in hip fracture patients was evaluated in other populations as well, which was variable compared to the prevalence we found in the current study. Ho et al. evaluated 239 Chinese hip fracture patients with a mean age of 82 years. Sarcopenia prevalence was 73.6% in males and 67.7% in females, when the diagnosis was based on relative appendicular SMM index and handgrip strength (according to the Asian Working Group for Sarcopenia (AWGS)), while the prevalence was 20.8% in males and 12.4% in females according to the EWGSOP definition [46]. In a study on 139 Taiwanese hip fracture patients with a mean age of 80.7 years, Chen et al. reported a sarcopenia prevalence of 50.4% according to the AWGS definitions. They reported significantly higher prevalence in males than females, 63.9% vs. 44.7%, respectively (*p* = 0.047); furthermore, in comparison with patients who did not have sarcopenia, sarcopenic patients were significantly older (83.5 ± 9.8 vs. 78 ± 8.8, p = 0.000), they had lower BMI (20.9 ± 3.3 vs. 24.3 ± 3.1, *p* = 0.000), lower handgrip strength (9.8 ± 5.4 vs. 13.8 ± 9.3, *p* = 0.007), and lower total body fat (31.5 ± 8.4 vs. 36.5 ± 6.4, *p* = 0.000) [44].

According to our univariate analysis, age and sex did not have a clear association with sarcopenia, while BMI, fat mass, BMR, and nutritional status had a significant negative association; on the other hand, the sum of SARC-F (≥ 4 points) had a significant positive association. After multivariate analysis, only protein levels and the sum of SARC-F remain significantly associated with sarcopenia. Furthermore, age was found to have a negative correlation with SMM and handgrip strength (*r* = −0.165, *p* = 0.056) and (*r* = −0.394, *p* < 0.001), respectively. Meanwhile, BMI positively correlated with SMM (*r* = 0.210, *p* = 0.014) and negatively correlated with handgrip strength (*r* = −0.165, *p* = 0.056). Ho et al. found a positive correlation between relative appendicular skeletal muscle mass index (RASM) and handgrip strength, body weight, hip BMD, BMI, and total fat mass [46]. Chen et al. reported that the RASM positively correlated with BMI, handgrip strength, and T-score in male patients, while in females, it only positively correlated with BMI and T-score. Furthermore, the authors reported that BMI was the only factor strongly correlated with RASM in males and females (*r* = 0.612 and 0.603, respectively) [44].

Our multivariate logistic regression analysis revealed that only the protein levels and SARC-F score remained significantly associated with sarcopenia, which could be explained by possible factors. Regarding the protein levels, the regression analysis results showed that higher protein levels are associated with significantly lower odds (95.6% reduction) of having sarcopenia. These results coincide with the normal biological pathway where skeletal muscle homeostasis relies on proper protein intake [56]. Furthermore, maintaining adequate protein levels through protein supplementation helps reduce muscle loss and synergistically improve sarcopenic patients'functional outcomes [57]. For the SARC-F Score, the regression analysis results showed that patients with a sum score of ≥ 4 are associated with about six times higher odds of having sarcopenia. For this score to remain a significant predictor even after multivariate regression analysis, it might be attributed to various factors. First, the screening nature of the score should inherently capture probable sarcopenic patients more than the confirmatory tests. Second, the nature of the score is dependent on collecting functional parameters related to patients'mobility and strength limitations, which might precede the actual measurable muscle loss [58]. Notably, 30.4% of sarcopenic patients in our study had SARC-F ≥ 4 (vs. 11.6% non-sarcopenic), suggesting that self-reported difficulties correlate with diagnostic criteria. This aligns with studies validating SARC-F as a screening tool in diverse populations, though its modest sensitivity underscores the need for confirmatory testing [59].

The incidence of fragility hip fracture in Egypt was estimated by the Egyptian Academy of Bone Health by assessing the data from a Fracture Liaison Service (FLS) National Register database over one year, they included patients > 40 years old, and the annual incidence of low energy hip fractures was 123.3 per 100,000 in women and 55.2 per 100,000 in men, furthermore, the authors reported significant differences according to geographical location northern vs. southern areas (the latter was the geographical location from where the current study data was collected) [28].

El-Miedany et al. reported a significant difference in BMD based on the geographic location, which might have affected the differences in fracture risk; furthermore, they highlighted the paramount role of modifiable risk factors in BMD-fracture relationship, where higher BMD was reported in population with greater height, weight, BMI, and lean mass [28]. In the current study, the nutritional status (total water, proteins, and minerals) and BMI were significantly lower in patients diagnosed with sarcopenia than in those without, which are considered modifiable risk factors amenable to correction even postoperatively. Moreover, correcting protein-energy malnutrition was linked to better recovery and functional outcomes in hip fracture patients [47, 48].

Unfortunately, evaluating why the results we obtained from the particular population included in our study differ from those obtained from other studies from Egypt and studies from different countries was not among our study aims; these could be attributed to the differences in socioeconomic conditions, pre-injury physical activity levels, dietary patterns, and health care access, including periodical check-up assessments. Furthermore, some reasons could explain why the incidence of sarcopenia in our population (southern Egypt) differs from the previous studies conducted on the Egyptian population but from different regions (northern areas). First, patients from southern Egypt are more involved in agriculture and manual labor due to their rural residences, which leads to less sedentary lifestyles and helps preserve muscle mass despite poor nutrition. Second, better community support due to the nature of extended family structures in rural southern Egypt might help older adults remain physically active (e.g., through household chores, walking, and religious activities), delaying functional decline.

Although the current study's major strength point was related to applying the EWGSOP2 definition for diagnosing sarcopenia in hip fracture patients from our population (Egyptian, North African), it also has some inherent Limitations: First, the sample size is considered small compared to previous studies, which might be attributed to the high selectivity of the inclusion criteria. To compensate for such an issue, we carried out a post-hoc power analysis for the differences between the primary outcomes, which indicated > 80% power for most variables, reinforcing the strength of the resulting associations. Second, the study population was limited to a specific geographical area (Southern Egypt), and the possible effects of geographical disparities and cultural and social factors were not thoroughly evaluated, which might limit the generalizability of the results. Furthermore, such high selectivity might be a source of selection bias. Third, we could not obtain a DXA scan assessment, which we clarified is related to some logistical and financial constraints. Fourth, we evaluated patients during the hospital stay only, and further follow up of those patients to document their functional and quality of life outcomes is paramount. Fifth, due to the study's cross-sectional nature, we could identify the factors associated with sarcopenia; however, we could not identify its predictors, which required a prospective observational study. Sixth, some evaluation tools, such as the Chair stands test (criterion 1 in EWGSOP2), Walk test, and Time up and Go test (criterion 3), were not feasible or applicable in hip fracture patients. Last, a longer follow up for the included patients is lacking, which helps in understanding the clinical outcomes differences between patients diagnosed with sarcopenia compared to those without.

*We have to allude to some clinical implications based on the current study findings:* First, raising awareness of the relatively high prevalence of sarcopenia in our patients who presented with low-energy trauma hip fractures will help early detection of sarcopenia (using a simple tool such as SARC-F questionnaire for screening, which proved a significant association with sarcopenia in our hip fracture patients, followed by the confirmatory tests if necessary) facilitating proper intervention, including, at least, nutritional support and dedicated rehabilitation programs leading eventually to better outcomes [45]. Second, as we found low protein levels to be a strongly associated factor with sarcopenia, nutritional supplementation, especially a rich protein diet in the postoperative period (which is correlated to better recovery [47, 48]), and spreading the importance of such a proper diet among the medical community in our area. Third, rehabilitation programs should be modified to improve patients'reduced muscle mass and strength by introducing more resistance training and encouraging early mobilization and weight bearing as tolerated. Last, there is a need for initiating a multidisciplinary team approach for hip fracture patients'care, as most hip fracture patients with associated sarcopenia might present with other medical comorbidities, which necessitates the involvement of different medical specialties (such as geriatrician, internist, nutritionist, and physiotherapist) for better care of those patients.

*Some of the challenges and their solutions* while implementing sarcopenia screening in our hip fracture population are worth noting: First, there is limited awareness of such a problem and its clinical and social impact, especially among our medical community. This issue could be addressed by integrating targeted educational programs related to sarcopenia, its diagnosis, and its consequences into medical training programs.

Second, there is a lack of training on screening sarcopenia in suspected patients using simple tools such as the SARC-F questionnaire; furthermore, it becomes more challenging for some hospitals to perform confirmatory tests (e.g., DXA scans) due to lacking required equipment. Possible solutions for this obstacle involve broad adoption and training on SARC-F questionnaire, especially as it showed significant association with sarcopenia in our patients, furthermore implementing further cheap and rapid screening tools such as inquiring about urinary incontinence (UI), where its presence showed a significant association with rectus abdominis muscle thickness (OR: 0.58; 95% CI 0.38–0.89; *p* = 0.01) according to Sahiner et al.; moreover, the authors reported that the overall sarcopenia risk per SARC-F score was significantly higher in patients with UI than those without (47.9% vs. %25.6, *p* = 0.03) [60]. The utility of UI assessment as an indicator of muscle mass loss was further confirmed in a study by Zhang and Li [61]; they also concluded that UI prevalence was significantly higher in sarcopenia patients, indicating a close association between UI and sarcopenia.

Third, patient compliance and understanding the importance of such screening rather than perceiving it as unnecessary cumbersome investigations. This could be resolved by initiating patient-targeted educational programs highlighting the importance of proper nutrition and alluding to some of the drawbacks of developing sarcopenia and the importance of their cooperation in filling out the questionnaires and carrying out the investigations. Furthermore, improving the hospital infrastructure by providing the necessary evaluation tools in one place and at an affordable cost for more patients'convenience.

Last is the burden of convincing higher authorities and stakeholders regarding the importance of integrating such screening programs as an integral part of hip fracture patients'care pathway and financially supporting such transformation. The best way to overcome this is by performing studies that show the cost-effectiveness of early sarcopenia diagnosis and management.

## Conclusion

We report a sarcopenia prevalence of 14.8% according to SARC-F scores, which increased to 17% when applying the operational definition of sarcopenia according to EWGSOP2 based on muscle mass and power testing among hip fracture adult patients admitted in our trauma service as a representative to Egyptian, and North African population. According to univariate analysis, BMI, fat mass, BMR, and nutritional status had a significant negative association with sarcopenia. Conversely, the sum of SARC-F (≥ 4 points) had a significant positive association. After multivariate analysis, only low protein levels and the sum of SARC-F remained the most robust independent associated factors with the presence of sarcopenia.

### Future directions

Based on our results, we believe that future research (prospective and, if possible, to be multicenter for including a more diverse population) is paramount to investigate the predictors of sarcopenia in hip fracture patients. Second, following up patients for more extended periods will provide details regarding the behavior of sarcopenia (improvement or deterioration) after surgical management and its effect on morbidity and mortality rates. Third, cost-effectiveness studies related to implementing sarcopenia screening and management protocols (especially in economically challenging environments such as our community) are highly recommended. Last, for a better understanding of sarcopenia and its associated factors, interventional studies should be carried out comparing various nutritional and rehabilitation protocols applied for such patients, aiming to develop a standardized management protocol for patients with sarcopenia associated with hip fractures.

## Supplementary Information

Supplementary file 1: STROBE Statement—Checklist of items that should be included in cross-sectional studies.

Supplementary file 2: Patients self-administered questionnaires to collect patients' basic demographic details.

Supplementary file 3: Patient habits, comorbidities, and nutritional factors associated with the risk of developing sarcopenia

## Data Availability

No datasets were generated or analysed during the current study.

## Abbreviations

EWGSOP2: European Working Group on Sarcopenia in Older People
AP: Anteroposterior
DSM-BIA: Direct segmental measurement bioelectrical impedance analysis
SMM: Skeletal muscle mass
BMI: Body mass index
OR: Odds ratio
DXA: Dual-energy X-ray absorptiometry

## References

[1] Giovannini S, Brau F, Forino R, Berti A, D'Ignazio F, Loreti C, Bellieni A, D'Angelo E, Di Caro F, Biscotti L, Coraci D, Fusco A, Padua L, Bernabei R (2021) Sarcopenia: Diagnosis and Management, State of the Art and Contribution of Ultrasound. J Clin Med 10 (23). 10.3390/jcm10235552 10.3390/jcm10235552 PMC8658070 34884255

[2] Cruz-Jentoft AJ, Bahat G, Bauer J, Boirie Y, Bruyere O, Cederholm T, Cooper C, Landi F, Rolland Y, Sayer AA, Schneider SM, Sieber CC, Topinkova E, Vandewoude M, Visser M, Zamboni M, Writing Group for the European Working Group on Sarcopenia in Older P, the Extended Group for E. Sarcopenia: revised European consensus on definition and diagnosis. Age Ageing. 2019;48(1):16–31. 10.1093/ageing/afy169. 30312372 10.1093/ageing/afy169 PMC6322506

[3] Cho MR, Lee S, Song SK. A Review of Sarcopenia Pathophysiology, Diagnosis, Treatment and Future Direction. J Korean Med Sci. 2022;37(18):e146. 10.3346/jkms.2022.37.e146. 35535373 10.3346/jkms.2022.37.e146 PMC9091430

[4] Cruz-Jentoft AJ, Sayer AA. Sarcopenia Lancet. 2019;393(10191):2636–46. 10.1016/S0140-6736(19)31138-9. 31171417 10.1016/S0140-6736(19)31138-9

[5] Narici MV, Maffulli N. Sarcopenia: characteristics, mechanisms and functional significance. Br Med Bull. 2010;95:139–59. 10.1093/bmb/ldq008. 20200012 10.1093/bmb/ldq008

[6] Prowse J, Jaiswal S, Gentle J, Sorial AK, Witham MD. Feasibility, acceptability and prognostic value of muscle mass and strength measurement in patients with hip fracture: a systematic review. Eur Geriatr Med. 2024;15(6):1603–14. 10.1007/s41999-024-01102-x. 39614068 10.1007/s41999-024-01102-x PMC11632060

[7] Cruz-Jentoft AJ, Landi F, Schneider SM, Zuniga C, Arai H, Boirie Y, Chen LK, Fielding RA, Martin FC, Michel JP, Sieber C, Stout JR, Studenski SA, Vellas B, Woo J, Zamboni M, Cederholm T. Prevalence of and interventions for sarcopenia in ageing adults: a systematic review. Report of the International Sarcopenia Initiative (EWGSOP and IWGS). Age Ageing. 2014;43(6):748–59. 10.1093/ageing/afu115. 25241753 10.1093/ageing/afu115 PMC4204661

[8] Mayhew AJ, Amog K, Phillips S, Parise G, McNicholas PD, de Souza RJ, Thabane L, Raina P. The prevalence of sarcopenia in community-dwelling older adults, an exploration of differences between studies and within definitions: a systematic review and meta-analyses. Age Ageing. 2019;48(1):48–56. 10.1093/ageing/afy106. 30052707 10.1093/ageing/afy106

[9] Saez-Lopez P, Branas F, Sanchez-Hernandez N, Alonso-Garcia N, Gonzalez-Montalvo JI. Hip fracture registries: utility, description, and comparison. Osteoporos Int. 2017;28(4):1157–66. 10.1007/s00198-016-3834-x. 27872956 10.1007/s00198-016-3834-x

[10] Huang P, Luo K, Xu J, Huang W, Yin W, Xiao M, Wang Y, Ding M, Huang X. Sarcopenia as a Risk Factor for Future Hip Fracture: A Meta-Analysis of Prospective Cohort Studies. J Nutr Health Aging. 2021;25(2):183–8. 10.1007/s12603-020-1474-5.33491032 10.1007/s12603-020-1474-5

[11] Chen M, Li Y, Yang Y, Zhuang W. Analysis of the risk factors for contralateral refracture after hip fracture surgery in elderly individuals: a retrospective study. J Orthop Surg Res. 2024;19(1):681. 10.1186/s13018-024-05177-x. 39438923 10.1186/s13018-024-05177-x PMC11515634

[12] Liu T, Zhang X, Zhang J, Ye P, Yang M, Tian M. Effect of the orthogeriatric co-management on older hip fracture patients with multimorbidity: a post-hoc exploratory subgroup analysis of a non-randomised controlled trial. J Orthop Surg Res. 2024;19(1):780. 10.1186/s13018-024-05263-0. 39574198 10.1186/s13018-024-05263-0 PMC11580192

[13] Johnell O, Kanis JA. An estimate of the worldwide prevalence and disability associated with osteoporotic fractures. Osteoporos Int. 2006;17(12):1726–33. 10.1007/s00198-006-0172-4. 16983459 10.1007/s00198-006-0172-4

[14] Gullberg B, Johnell O, Kanis JA. World-wide projections for hip fracture. Osteoporos Int. 1997;7(5):407–13. 10.1007/pl00004148. 9425497 10.1007/pl00004148

[15] El Miedany Y, El Gaafary M, Gadallah N, Mahran S, Fathi N, Abu Zaid MH, Tabra SAH, Hassan W, Elwakil W. Osteoporosis treatment gap in patients at risk of fracture in Egypt: a multi-center, cross-sectional observational study. Arch Osteoporos. 2023;18(1):58. 10.1007/s11657-023-01252-8. 37127804 10.1007/s11657-023-01252-8

[16] Liu E. Hip fractures: mortality, economic burden, and organisational factors for improved patient outcomes. Lancet Healthy Longev. 2023;4(8):e360–1. 10.1016/S2666-7568(23)00102-2. 37442153 10.1016/S2666-7568(23)00102-2

[17] Di Monaco M, Castiglioni C, Bardesono F, Milano E, Massazza G. Sarcopenia, osteoporosis and the burden of prevalent vertebral fractures: a cross-sectional study of 350 women with hip fracture. Eur J Phys Rehabil Med. 2020;56(2):184–90. 10.23736/S1973-9087.20.05991-2. 32052946 10.23736/S1973-9087.20.05991-2

[18] Han X, Han L, Chu F, Liu B, Song F, Jia D, Wang H. Predictors for 1-year mortality in geriatric patients following fragile intertrochanteric fracture surgery. J Orthop Surg Res. 2024;19(1):701. 10.1186/s13018-024-05219-4. 39472932 10.1186/s13018-024-05219-4 PMC11523668

[19] Huang J, Lin L, Lyu J, Fang X, Zhang W. Hip arthroplasty following failure of internal fixation in intertrochanteric femoral fractures: classification decision-making for femoral stem selection and clinical validation. J Orthop Surg Res. 2024;19(1):671. 10.1186/s13018-024-05136-6. 39425202 10.1186/s13018-024-05136-6 PMC11490114

[20] Yeung SSY, Reijnierse EM, Pham VK, Trappenburg MC, Lim WK, Meskers CGM, Maier AB. Sarcopenia and its association with falls and fractures in older adults: A systematic review and meta-analysis. J Cachexia Sarcopenia Muscle. 2019;10(3):485–500. 10.1002/jcsm.12411. 30993881 10.1002/jcsm.12411 PMC6596401

[21] Zhang X, Huang P, Dou Q, Wang C, Zhang W, Yang Y, Wang J, Xie X, Zhou J, Zeng Y. Falls among older adults with sarcopenia dwelling in nursing home or community: A meta-analysis. Clin Nutr. 2020;39(1):33–9. 10.1016/j.clnu.2019.01.002. 30665817 10.1016/j.clnu.2019.01.002

[22] Zhang Y, Hao Q, Ge M, Dong B. Association of sarcopenia and fractures in community-dwelling older adults: a systematic review and meta-analysis of cohort studies. Osteoporos Int. 2018;29(6):1253–62. 10.1007/s00198-018-4429-5. 29500527 10.1007/s00198-018-4429-5

[23] Dionyssiotis Y, de Leon AO. Sarcopenia and Hip Fractures. J Frailty Sarcopenia Falls. 2024;9(1):1–3. 10.22540/JFSF-09-001. 38444544 10.22540/JFSF-09-001 PMC10910255

[24] Beaudart C, Demonceau C, Reginster JY, Locquet M, Cesari M, Cruz Jentoft AJ, Bruyere O. Sarcopenia and health-related quality of life: A systematic review and meta-analysis. J Cachexia Sarcopenia Muscle. 2023;14(3):1228–43. 10.1002/jcsm.13243. 37139947 10.1002/jcsm.13243 PMC10235892

[25] Veronese N, Koyanagi A, Cereda E, Maggi S, Barbagallo M, Dominguez LJ, Smith L. Sarcopenia reduces quality of life in the long-term: longitudinal analyses from the English longitudinal study of ageing. Eur Geriatr Med. 2022;13(3):633–9. 10.1007/s41999-022-00627-3. 35212911 10.1007/s41999-022-00627-3 PMC9151534

[26] El Miedany Y, El Gaafary M, Gadallah N, Mahran S, Hassan W, Fathi N, Abu-Zaid MH, Tabra SAA, Shalaby RH, Elwakil W. Targeted optimum care approach for osteoporotic fragility fractures: tailored strategy based on risk stratification to reduce incidents of falls-an initiative by the Egyptian Academy of bone health based on the FLS national register. Arch Osteoporos. 2023;18(1):139. 10.1007/s11657-023-01347-2. 37985519 10.1007/s11657-023-01347-2

[27] Sanad HT, Hamza SA, Metwaly RG, Elbehery HM, RMS EL,. Sarcopenia and Related Functional Outcomes Following Hip Surgery Among Egyptian Geriatric Patients With Hip Fracture. Cureus. 2023;15(8):e43166. 10.7759/cureus.43166. 37692743 10.7759/cureus.43166 PMC10484563

[28] El Miedany Y, El Gaafary M, Gadallah N, Mahran S, Fathi N, Abu-Zaid MH, Tabra SAA, Shalaby RH, Abdelrafea B, Hassan W, Farouk O, Nafady M, Ibrahim SIM, Ali MA, Elwakil W. Incidence and geographic characteristics of the population with osteoporotic hip fracture in Egypt- by the Egyptian Academy of Bone Health. Arch Osteoporos. 2023;18(1):115. 10.1007/s11657-023-01325-8. 37688741 10.1007/s11657-023-01325-8

[29] von Elm E, Altman DG, Egger M, Pocock SJ, Gotzsche PC, Vandenbroucke JP, Initiative S. The Strengthening the Reporting of Observational Studies in Epidemiology (STROBE) statement: guidelines for reporting observational studies. J Clin Epidemiol. 2008;61(4):344–9. 10.1016/j.jclinepi.2007.11.008. 18313558 10.1016/j.jclinepi.2007.11.008

[30] Hida T, Ishiguro N, Shimokata H, Sakai Y, Matsui Y, Takemura M, Terabe Y, Harada A. High prevalence of sarcopenia and reduced leg muscle mass in Japanese patients immediately after a hip fracture. Geriatr Gerontol Int. 2013;13(2):413–20. 10.1111/j.1447-0594.2012.00918.x. 22816427 10.1111/j.1447-0594.2012.00918.x

[31] Abdelnasser MK, Khalifa AA, Amir KG, Hassan MA, Eisa AA, El-Adly WY, Ibrahim AK, Farouk OA, Abubeih HA. Mortality incidence and its determinants after fragility hip fractures: a prospective cohort study from an Egyptian level one trauma center. Afr Health Sci. 2021;21(2):806–16. 10.4314/ahs.v21i2.41. 34795739 10.4314/ahs.v21i2.41 PMC8568210

[32] Mahran DG, Farouk O, Ismail MA, Alaa MM, Eisa A, Ragab II. Effectiveness of home based intervention program in reducing mortality of hip fracture patients: A non-randomized controlled trial. Arch Gerontol Geriatr. 2019;81:8–17. 10.1016/j.archger.2018.11.007. 30471472 10.1016/j.archger.2018.11.007

[33] Malmstrom TK, Morley JE. SARC-F: a simple questionnaire to rapidly diagnose sarcopenia. J Am Med Dir Assoc. 2013;14(8):531–2. 10.1016/j.jamda.2013.05.018. 23810110 10.1016/j.jamda.2013.05.018

[34] Malmstrom TK, Morley JE. Sarcopenia: The Target Population. J Frailty Aging. 2013;2(1):55–6. 10.14283/jfa.2013.8. 27070457 10.14283/jfa.2013.8

[35] Yu SC, Khow KS, Jadczak AD, Visvanathan R. Clinical Screening Tools for Sarcopenia and Its Management. Curr Gerontol Geriatr Res. 2016;2016:5978523. 10.1155/2016/5978523. 26966433 10.1155/2016/5978523 PMC4757731

[36] D’Adamo CR, Hawkes WG, Miller RR, Jones M, Hochberg M, Yu-Yahiro J, Hebel JR, Magaziner J. Short-term changes in body composition after surgical repair of hip fracture. Age Ageing. 2014;43(2):275–80. 10.1093/ageing/aft198. 24370941 10.1093/ageing/aft198 PMC3927774

[37] McLester CN, Nickerson BS, Kliszczewicz BM, McLester JR. Reliability and Agreement of Various InBody Body Composition Analyzers as Compared to Dual-Energy X-Ray Absorptiometry in Healthy Men and Women. J Clin Densitom. 2020;23(3):443–50. 10.1016/j.jocd.2018.10.008. 30472111 10.1016/j.jocd.2018.10.008

[38] Schubert MM, Seay RF, Spain KK, Clarke HE, Taylor JK. Reliability and validity of various laboratory methods of body composition assessment in young adults. Clin Physiol Funct Imaging. 2019;39(2):150–9. 10.1111/cpf.12550. 30325573 10.1111/cpf.12550

[39] Peterson CM, Thomas DM, Blackburn GL, Heymsfield SB. Universal equation for estimating ideal body weight and body weight at any BMI. Am J Clin Nutr. 2016;103(5):1197–203. 10.3945/ajcn.115.121178. 27030535 10.3945/ajcn.115.121178 PMC4841935

[40] Podell K. Hand Dynamometer. In: Kreutzer JS, DeLuca J, Caplan B (eds) Encyclopedia of Clinical Neuropsychology. Springer New York, New York, NY. 2011. pp 1208–1209. 10.1007/978-0-387-79948-3_190

[41] Ibrahim K, May CR, Patel HP, Baxter M, Sayer AA, Roberts HC. Implementation of grip strength measurement in medicine for older people wards as part of routine admission assessment: identifying facilitators and barriers using a theory-led intervention. BMC Geriatr. 2018;18(1):79. 10.1186/s12877-018-0768-5. 29566673 10.1186/s12877-018-0768-5 PMC5865333

[42] Kim JH, Lim S, Choi SH, Kim KM, Yoon JW, Kim KW, Lim JY, Park KS, Jang HC. Sarcopenia: an independent predictor of mortality in community-dwelling older Korean men. J Gerontol A Biol Sci Med Sci. 2014;69(10):1244–52. 10.1093/gerona/glu050. 24721723 10.1093/gerona/glu050

[43] da Silva AT, de Oliveira Duarte YA, Ferreira Santos JL, Wong R, Lebrao ML. Sarcopenia according to the european working group on sarcopenia in older people (EWGSOP) versus Dynapenia as a risk factor for disability in the elderly. J Nutr Health Aging. 2014;18(5):547–53. 10.1007/s12603-014-0465-9.24886743 10.1007/s12603-014-0465-9

[44] Chen YP, Wong PK, Tsai MJ, Chang WC, Hsieh TS, Leu TH, Jeff Lin CF, Lee CH, Kuo YJ, Lin CY. The high prevalence of sarcopenia and its associated outcomes following hip surgery in Taiwanese geriatric patients with a hip fracture. J Formos Med Assoc. 2020;119(12):1807–16. 10.1016/j.jfma.2020.02.004. 32107098 10.1016/j.jfma.2020.02.004

[45] Chen YP, Kuo YJ, Hung SW, Wen TW, Chien PC, Chiang MH, Maffulli N, Lin CY. Loss of skeletal muscle mass can be predicted by sarcopenia and reflects poor functional recovery at one year after surgery for geriatric hip fractures. Injury. 2021;52(11):3446–52. 10.1016/j.injury.2021.08.007. 34404509 10.1016/j.injury.2021.08.007

[46] Ho AW, Lee MM, Chan EW, Ng HM, Lee CW, Ng WS, Wong SH. Prevalence of pre-sarcopenia and sarcopenia in Hong Kong Chinese geriatric patients with hip fracture and its correlation with different factors. Hong Kong Med J. 2016;22(1):23–9. 10.12809/hkmj154570. 26680156 10.12809/hkmj154570

[47] Dionyssiotis Y, Chhetri JK, Piotrowicz K, Gueye T, Sánchez E. Impact of nutrition for rehabilitation of older patients: Report on the 1st EICA-ESPRM-EUGMS Train the Trainers Course. European Geriatric Medicine. 2017;8(2):183–90. 10.1016/j.eurger.2016.11.011.

[48] Irisawa H, Mizushima T. Relationship between Nutritional Status, Body Composition, Muscle Strength, and Functional Recovery in Patients with Proximal Femur Fracture. Nutrients. 2022; 14 (11). 10.3390/nu14112298 10.3390/nu14112298 PMC9183158 35684096

[49] Karlsson M, Nilsson JA, Sernbo I, Redlund-Johnell I, Johnell O, Obrant KJ. Changes of bone mineral mass and soft tissue composition after hip fracture. Bone. 1996;18(1):19–22. 10.1016/8756-3282(95)00422-x. 8717532 10.1016/8756-3282(95)00422-x

[50] Reider L, Owen EC, Dreyer HC, Fitton LS, Willey MC, Metrc,. Loss of Muscle Mass and Strength After Hip Fracture: an Intervention Target for Nutrition Supplementation. Curr Osteoporos Rep. 2023;21(6):710–8. 10.1007/s11914-023-00836-0. 38019345 10.1007/s11914-023-00836-0

[51] Fox KM, Magaziner J, Hawkes WG, Yu-Yahiro J, Hebel JR, Zimmerman SI, Holder L, Michael R. Loss of bone density and lean body mass after hip fracture. Osteoporos Int. 2000;11(1):31–5. 10.1007/s001980050003. 10663356 10.1007/s001980050003

[52] Cheng L, Wang S. Correlation between bone mineral density and sarcopenia in US adults: a population-based study. J Orthop Surg Res. 2023;18(1):588. 10.1186/s13018-023-04034-7. 37559054 10.1186/s13018-023-04034-7 PMC10410911

[53] Oyama K, Ogawa H, Osaki M, Kono S, Hasegawa M. Izutani Y Validation of InBody 770 as a Tool for Assessing Skeletal Muscle Mass. Clin Surg. 2019;4:2637.

[54] Branco MG, Mateus C, Capelas ML, Pimenta N, Santos T, Makitie A, Ganhao-Arranhado S, Trabulo C, Ravasco P. Bioelectrical Impedance Analysis (BIA) for the Assessment of Body Composition in Oncology: A Scoping Review. Nutrients. 2023;15 (22). 10.3390/nu15224792 10.3390/nu15224792 PMC10675768 38004186

[55] Macedo S, de Souza Macedo PR, Barbosa WS, Maciel ACC. Use of the Ishii Test for screening sarcopenia in older adults: a systematic review with meta-analysis of diagnostic test accuracy (DTA) studies. BMC Geriatr. 2024;24(1):609. 10.1186/s12877-024-05155-2. 39014328 10.1186/s12877-024-05155-2 PMC11253494

[56] Deldicque L. Protein Intake and Exercise-Induced Skeletal Muscle Hypertrophy: An Update. Nutrients. 2020; 12 (7). 10.3390/nu12072023 10.3390/nu12072023 PMC7400877 32646013

[57] Jang YJ. The Effects of Protein and Supplements on Sarcopenia in Human Clinical Studies: How Older Adults Should Consume Protein and Supplements. J Microbiol Biotechnol. 2023;33(2):143–50. 10.4014/jmb.2210.10014. 36474318 10.4014/jmb.2210.10014 PMC9998208

[58] Malmstrom TK, Miller DK, Simonsick EM, Ferrucci L, Morley JE. SARC-F: a symptom score to predict persons with sarcopenia at risk for poor functional outcomes. J Cachexia Sarcopenia Muscle. 2016;7(1):28–36. 10.1002/jcsm.12048. 27066316 10.1002/jcsm.12048 PMC4799853

[59] Voelker SN, Michalopoulos N, Maier AB, Reijnierse EM. Reliability and Concurrent Validity of the SARC-F and Its Modified Versions: A Systematic Review and Meta-Analysis. J Am Med Dir Assoc. 2021;22 (9):1864–1876 e1816. 10.1016/j.jamda.2021.05.011 10.1016/j.jamda.2021.05.011 34144049

[60] Sahiner Z, Mangir N, Guner M, Ceylan S, Hafizoglu M, Karaduman D, Atbas C, Bas AO, Ozer YP, Balci C, Dogu BB, Halil M, Cankurtaran M. The relationship between urinary incontinence and abdominal muscle thickness in community-dwelling older women undergoing comprehensive geriatric assessment. Eur Geriatr Med. 2023;14(6):1319–25. 10.1007/s41999-023-00874-y. 37837573 10.1007/s41999-023-00874-y

[61] Zhang F, Li W. Association of Sarcopenia and Urinary Incontinence in Adult Women Aged Less Than 60 years. Int J Womens Health. 2025;17:695–709. 10.2147/IJWH.S516752. 40070684 10.2147/IJWH.S516752 PMC11895681

