# Supplementary material for: Sarcopenia and its associated factors among hip fracture patients admitted to a North African (Egyptian) Level one trauma center, a cross-sectional study
Source: J Orthop Surg Res. 2025 May 13;20:459. doi: 10.1186/s13018-025-05841-w (PMC12070717; doi:10.1186/s13018-025-05841-w)
Supplement: Supplementary file 2 — Patients self-administered questionnaires to collect patients' basic demographic details. [file 13018_2025_5841_MOESM2_ESM.pdf]

كلية الطب

دراسة عن كسر عظمة الفخذ الناتج عن هشاشة العظام بمستشفى أسيوط الجامعي

تاريخ دخول المستشفى:

رقم التذكرة:

العنوان التفصيلي:

أرقام التليفونات:

1	اسم المريض: .... | 2 | الرقم:	
3	السن: .... | 4 | النوع: ذكر ( ) آنثي( )	
5	التعليم: 1) أمية 2) يقرأ وتكتب 3) ابتدائى 4) إعدادى 5) ثانوى 6) جامعى	
6	الوظيفة: 1) ربة منزل 2)فلاح 3) عامل حرفي 4) عامل غير حرفي 5)موظف قطاع خاص 6) موظف قطاع عام 7) عمل حر 8) متخصص 9) لا يعمل 10) بالمعاش 5) أخرى (تذكر ....)	
	**أسئلة خاصة بالمريضات**:	
	**تاريخ الدورة الشهرية**	
7	السن عند بداية الدورة الشهرية: .... سنة	
8	هل كانت الدورة الشهرية منتظمة؟ (21- 35 يوما) 1) نعم 2) لا 3) شبه منتظمة	
9	السن عند بداية الإنجاب: .... سنة	
10	عدد مرات الحمل:	
11	منذ متى انقطعت عنك الدورة	
12	سبب سن اليأس: 1) طبيعى 2) جراحى 3) مبكر(قبل ٤٥)	
	**الرضاعة الطبيعية**:	
13	عدد الأطفالالذين تم ارضاعهم طبيعيا.............	
14	فترة الرضاعة الكلية:	

**عادات شخصية وغذائية**:		
28	**التدخين:** 1) تدخن 2) مدخنة سابقة 3) لا تدخن	
29	نوع التدخين: 1) سجائر 2) جوزة 3) الاثنين	
30	كم مرة يوميا:	
31	الفترة:	
32	**شرب القهوة:** 1) منتظم 2) احيانا 3) لا	
33	عدد مرات شرب القهوة يوميا:	
34	**شرب الشاى:** 1) منتظم 2) احيانا 3) لا	
35	عدد مرت شرب القهوة يوميا:	
36	**المياه الغازية:** 1) منتظم 2) أحيانا 3) لا	
37	عد مرات شرب المياه لغازية يوميا:	
38	**شرب اللبن:** –لا<br>–نعم............يوميا؟ اسبوعيا؟	
39	**أكل الجبنة: _لا**<br>_ نعم............يوميا؟ اسبوعيا؟	
40	**أكل الزبادي: : _لا**<br>_ نعم............يوميا؟ اسبوعيا؟	
41	**فترة لتعرض للشمسً** فى أغلب الأيام: .... دقيقة	

النشاط الحركي

	السؤال	الإجابة	الكود
في العمل(شغل تقيل ولا متوسط؟؟)			
42	هل طبيعة شغلك فيه مجهود كبير بيخليك تنهت وقلبك يرفرف عليك؟ (شيل حاجات تقيلة مثلا)	نعم (1) لا(2) اذهب ل P4	P1
43	خلال الأسبوع : كام يوم بيكون الشغل فيه تقيل وعاوز مجهود؟	عدد الأيام	P2
44	يوم الشغل اللي بيكون تقيل: بتعمل مجهود كام ساعة؟	ساعة: دقيقة:	P3
45	هل طبيعة شغلك عبارة عن مجهود متوسط؟ (شيل حاجات خفيفة او مشي سريع)	نعم (1) لا(2) اذهب ل P7	P4
46	خلال الأسبوع:كام يوم بتشتغل فيه الشغل المتوسط ده؟	عدد الأيام	P5

47	يوم الشغل اللي بيكون تقيل: بتعمل مجهود كام ساعة؟	ساعة: دقيقة:	P6
التنقل			
48	علشان تروح من مكان لمكان: بتتمشي ؟؟(مدة 10 دقائق ع الأقل)	نعم (1) لا(2) اذهب ل P10	P7
49	خلال الأسبوع : كام يوم بتتمشى فيهم بين الأماكن؟	عدد الأيام	P8
50	في اليوم الواحد: قد ايه الوقت اللي بتقضيه في المشي	ساعة: دقيقة:	P9
النشاط و الرياضة في وقت الفراغ...خارج العمل (عنيفة ولا خفيفة)			
51	هل بتلعب رياضة عنيفة(كرة قدم او جري سريع)؟	نعم (1) لا(2) اذهب ل P13	P10
52	خلال الأسبوع : كام يوم بتلعب فيهم رياضة؟	عدد الأيام	P11
53	لما بتلعب رياضة: بتلعب كام ساعة؟	ساعة: دقيقة:	P12
54	هل بتلعب رياضة خفيفة(مشي بسيط او كرة طايرة او سباحة بسيطة)؟	نعم (1) لا(2) اذهب ل P16	P13
55	خلال الأسبوع : كام يوم بتلعب فيهم رياضة؟	عدد الأيام	P14
56	لما بتلعب رياضة: بتلعب كام ساعة؟	ساعة: دقيقة:	P15
الأنشطة خلال الجلوس وليس النوم			
57	كام ساعة في اليوم بتعمل فيها حاجات مش محتاجة حركة(تتفرج ع التلفزيون..قراية..قعدة مع صحابك..قاعد في مواصلة)	ساعة: دقيقة:	P16

**التاريخ المرضى**:		
58	هل عانيت من كسر له علاقة بالهشاشة فى أى جزء من الجسم؟ 1) نعم 2) لا	
59	**فى حالة نعم:** نوعه:	
60	السن عند حدوث الكسر:	
61	كيف تم علاجه؟ 1) جراحياً 2) جبيرة	
62	المدة التى استغرقها العلاج:	
63	تأثير الكسر: 1) الاعتماد على النفس 2) الاعتماد على الغير 3) الحجز فى المستشفى لفترة طويلة4) الاعتماد على عكاز أو جهاز طبى لفترة طويلة	
64	أى شكاوى أخرى:	
65	**سكر الدم:** 1) نعم 2) لا	

66	**نوع السكر:** 1) النوع الأول (سن صغير) 2) النوع الثانى (سن كبير)	
67	فترة المرض:	
68	**أمراض عصبية تسببت فى قلة الحركة (الشلل النصفى... الخ)** 1) نعم 2) لا	
69	فترة المرض:	
70	التهاب رئوي 1) نعم 2) لا	
71	فترة المرض:	
72	أمراض الجهاز التنفسي COPD 1) نعم 2) لا	
73	فترة المرض:	
74	جلطة في القلب أو ذبحة 1) نعم 2) لا	
75	فترة المرض:	
76	هبوط في القلب 1) نعم 2) لا	
77	فترة المرض:	
78	سكتة دماغية أو جلطة في المخ 1) نعم 2) لا	
79	فترة المرض:	
80	إلتهابات في المسالك البولية 1) نعم 2) لا	
81	فترة المرض:	
82	سرطانات 1) نعم 2) لا	
83	فترة المرض:	
84	**أمراض مفاصل تسببت فى قلة الحركة (خشونة الركبة... الخ)** 1) نعم 2) لا	
85	فترة المرض:	
86	**فشل كلوى:** 1) نعم 2) لا	
87	فترة المرض:	
88	**ارتفاع ضغط الدم:** 1) نعم 2) لا	
89	فترة المرض:	
90	**فشل كبدي:** 1) نعم 2) لا	
91	فترة المرض:	
92	**حساسية الصدر:** 1) نعم 2) لا	
93	فترة المرض:	
94	**روماتويد مفاصل:** 1) نعم 2) لا	
95	فترة المرض:	
96	**زيادة هرمون الغدة الدرقية:** 1) نعم 2) لا	
97	فترة المرض:	
98	**زيادة هرمون الغدة الجاردرقية:** 1) نعم 2) لا	
99	فترة المرض:	
100	**أنوركسيا نرفوزا:** 1) نعم 2) لا	
101	فترة المرض:	
102	**عمليات فى المعدة (استئصال جزء):** 1) نعم 2) لا	
103	فترة المرض:	

104	**أمراض سوء الهضم:** 1) نعم 2) لا	
105	فترة المرض:	
106	**مرض الذئبة الحمراء:** 1) نعم 2) لا	
107	فترة المرض:	
108	**أمراض جلدية مزمنةتعالج بالكورتيزون:** 1) نعم 2) لا	
109	تذكر الأمراض الجلدية (..)	
110	فترة المرض:	
111	**اكتئاب:** 1) نعم 2) لا	
112	فترة المرض:	
113	**مرض الصرع؟** 1) نعم 2) لا	
114	فترة المرض:	
115	**شلل رعاش؟** 1) نعم 2) لا	
116	فترة المرض:	
117	أمراض أخرى (تذكر ....)	
	**التاريخ العلاجى**:	
118	هرمونات تعويضية (استروجين- بروجستيرون): 1) نعم 2) لا	
119	فترة العلاج:	
120	الكورتيزون: 1) نعم 2) لا	
121	فترة العلاج:	
122	مدرات البول (لازكس): 1) نعم 2) لا	
123	فترة العلاج:	

124	مضادات حموضة تحتوى على الألمونيوم: 1) نعم 2) لا	
125	فترة العلاج:	
126	مضادات حموضة أخرى: سيمتيدين (زانتاك): 1) نعم 2) لا	
127	فترة العلاج:	
128	مضادات التشنج (الصرع): 1) نعم 2) لا	
129	فترة العلاج:	
130	أدوية السرطان (الكيماوية): 1) نعم 2) لا	
131	فترة العلاج:	
132	هرمون الغدة الدرقية: 1) نعم 2) لا	
133	فترة العلاج:	
134	هيبارين: 1) نعم 2) لا	
135	فترة العلاج:	
136	سيكلوسبورين (أ): 1) نعم 2) لا	
137	فترة العلاج:	
138	تاموكسفين: 1) نعم 2) لا	
139	فترة العلاج:	
**التاريخ الجراحي**:		
140	استئصال رحم؟ 1) نعم 2) لا	
141	السن عند إجراء العملية؟ .... سنة	
142	استئصال مبايض؟ 1) نعم 2) لا	
143	السن عند إجراء العملية؟ .... سنة	
144	جراحة فى الغدة الدرقية؟ 1) نعم 2) لا	
145	السن عند إجراء العملية؟ .... سنة	
**الفحص:**		
146	الوزن: .... كجم	
147	الطول: .... سم	

تحاليل المريض(كل رابع مريض)

Vit. D		
Ca		
Po4		
ALP		

Peri operative assessment

**Preoperative**:		
Fracture		
Ao code:		
**DM**	Yes ■	No ■
Mech. Of trauma	High energy ■	Low energy ■
Decision:		
Prev. Fr.	Yes ■	No ■
ttt prev. Fr.		
**Side**		
**Other injuries**		
**Anti thrombotic ttt**	Yes ■	No ■
**If yes what?**		

**Intraoperative**		
Type of op.		
Surgeon :	Name	Rank
**Op. site**		
**Anaethesia**	Regional ■	General ■
**Position**		
**Blood loss**		
**Intraop. Image**	Yes ■	No ■
**Duration**		
**Compl.**	Yes ■	No ■
**If yes what.....**		
**Drain**	Yes ■	No ■
**Bl. Transfusion**	Yes ■	No ■

Postoperative		
**Dexa**	T-score:	
**Sing index**	I ■ II ■ III ■ IV ■ V ■	
**Hospital stay**		
**Post. Op compl.**	Yes ■	No ■
If yes what		
**Antiporosisttt**	Yes ■	No ■
If yes what		
**Follow up**		
