# Supplementary material for: Sarcopenia and its associated factors among hip fracture patients admitted to a North African (Egyptian) Level one trauma center, a cross-sectional study
Source: J Orthop Surg Res. 2025 May 13;20:459. doi: 10.1186/s13018-025-05841-w (PMC12070717; doi:10.1186/s13018-025-05841-w)
Supplement: Supplementary file 3 — Patient habits, comorbidities, and nutritional factors associated with the risk of developing sarcopenia [file 13018_2025_5841_MOESM3_ESM.docx]

| Supplementary file 3: Patient habits, comorbidities, and nutritional factors associated with the risk of developing sarcopenia | | | | |
| --- | --- | --- | --- | --- |
| **Variable name** | | **No Sarcopenia (n=112)** | **Sarcopenia (n=23)** | **P value**^¶^ |
|  |  |  |  |  |
| Smoking | No | 90 (80.4) | 17 (73.9) | 0.572 |
|  | Yes | 22 (19.6) | 6 (26.1) |  |
| Fracture type | Trochanteric fracture | 67 (59.8) | 13 (56.5) | 0.769 |
|  | Neck of femur | 45 (40.2) | 10 (43.5) |  |
| Steroid intake | No | 108 (96.4) | 22 (95.7) | 1 |
|  | Yes | 4 (3.6) | 1 (4.3) |  |
| Chronic diseases | No | 93 (83.0) | 15 (65.2) | 0.082 |
|  | Yes | 19 (17.0) | 8 (34.8) |  |
|  |  |  |  |  |
| **Diabetes mellitus** | No | 83 (74.1) | 16 (69.6) | 0.654 |
|  | Yes | 29 (25.9) | 7 (30.4) |  |
| **Hypertension** | No | 75 (67.0) | 15 (65.2) | 0.871 |
|  | Yes | 37 (33.0) | 8 (34.8) |  |
| **nutritional habits** | | | | |
| **Coffee consumption** | No | 104 (92.9) | 21 (91.3) | 0.679 |
|  | Yes | 8 (7.1) | 2 (8.7) |  |
| **Tea consumption** | No | 18 (16.1) | 6 (26.1) | 0.246 |
|  | Yes | 94 (83.9) | 17 (73.9) |  |
| **Soda consumption** | No | 93 (83.0) | 17 (73.9) | 0.375 |
|  | Yes | 19 (17.0) | 6 (26.1) |  |
| **Milk** | No | 81 (72.3) | 16 (69.6) | 0.789 |
|  | Yes | 31 (27.7) | 7 (30.4) |  |
| **Cheese** | No | 82 (73.2) | 15 (65.2) | 0.437 |
|  | Yes | 30 (26.8) | 8 (34.8) |  |
| **Yogurt** | No | 93 (83.0) | 19 (82.6) | 1 |
|  | Yes | 19 (17.0) | 4 (17.4) |  |
| Data are presented as number (percentage). Significance defined by P-value ≤ 0.05.  ¶ Chi square (χ2) test and Fisher Exact test were used for comparison. | | | | |
